# Q-AVOA-net: A multi-domain, quantum-inspired feature-selection and deep-fuzzy framework for interpretable white blood cell classification

**DOI:** 10.1016/j.isci.2026.116507

**Published:** 2026-09-02

**Authors:** Omid Eslamifar, Mohammadreza Soltani, Seyed Mohammad Jalal Rastegar Fatemi

**Affiliations:** 1 Department of Electrical Engineering, Saveh Branch, Islamic Azad University, Saveh, Iran; 2 Department of Electrical Engineering, Kho.C, Islamic Azad University, Khomeinishahr, Iran

**Keywords:** white blood cell classification, quantum-inspired optimization, African vulture, optimization algorithm, contourlet transform, deep fuzzy clustering, medical image analysis, explainable AI, interpretable deep learning

## Abstract

Reliable white blood cell (WBC) classification from peripheral blood smear microscopy is hindered by stain variability, domain shift, class imbalance, and limited interpretability. We propose Q-AVOA-Net, a multi-domain and clinician-oriented framework integrating Enhanced Contourlet Transform and a Learnable Gabor Filter Bank for structure-texture encoding, Bi-LSTM attention for dependency modeling, Q-AVOA for multi-objective feature selection, and an interpretable fuzzy decision layer that outputs class-wise membership evidence. Evaluated on Raabin-WBC, LISC, and BCCD with cross-dataset and robustness testing, Q-AVOA-Net achieves 97.2 ± 0.2% accuracy on the integrated evaluation framework (AUC 0.988) and improves confidence reliability (ECE 0.018). The selected representation is compact (1280 features) while maintaining efficient inference (20.7 ms/image). Together, these results support robust, calibrated, and interpretable WBC classification for microscopy-based decision support.

## Highlights

• Q-AVOA-Net fuses multi-domain features, quantum selection, and fuzzy interpretability
• The model generalizes robustly across datasets and image perturbations
• Q-AVOA stabilizes feature selection for compact, efficient representations
• Fuzzy membership scores support clinician-oriented, uncertainty-aware triage

## Graphical abstract

**Figure:**
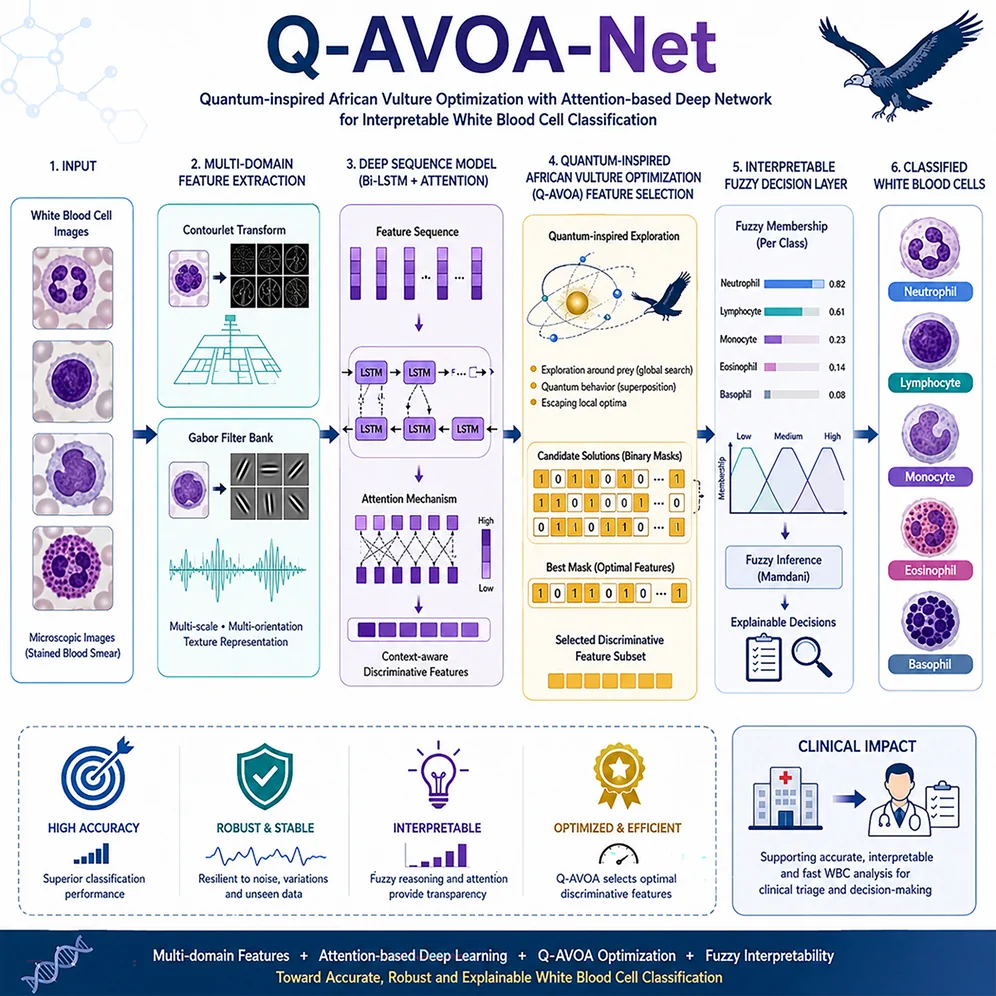

## Teaser

Image-guided intervention; Biomedical Engineering

## Introduction

Accurate identification and subtype classification of white blood cells (WBCs) from peripheral blood smear microscopy is fundamental to hematological diagnosis and disease monitoring, supporting clinical decision-making in infections, inflammatory disorders, and hematologic malignancies. In routine workflows, differential WBC counting is still largely performed via manual microscopic inspection. While effective, manual review is labor-intensive and time-consuming, and it is susceptible to inter- and intra-observer variability, particularly under subtle morphological differences, variable staining quality, and the presence of rare subtypes. These constraints have motivated automated WBC classification systems that aim to improve throughput and diagnostic consistency without compromising clinical interpretability.

Deep learning has driven major progress in microscopy image analysis, with convolutional neural networks (CNNs) and attention-based architectures achieving strong performance on benchmark datasets. However, several barriers continue to limit reliable real-world deployment. First, microscopy images exhibit domain shift across laboratories due to differences in staining protocols, slide preparation, illumination, and imaging equipment, which can degrade generalization when models are trained and tested under mismatched acquisition conditions. Second, WBC datasets are frequently class-imbalanced, and performance on rare subtypes is often under-characterized despite its clinical importance; for screening applications, rare-class false negatives can be disproportionately costly. Third, high-capacity models and multi-stage pipelines may incur substantial computational cost, not only at inference but also during training when additional preprocessing or feature extraction stages are introduced. Finally, many high-performing methods remain partially opaque, and their probability outputs may be poorly calibrated, limiting clinical trust and practical usability.

**Figure 1:**
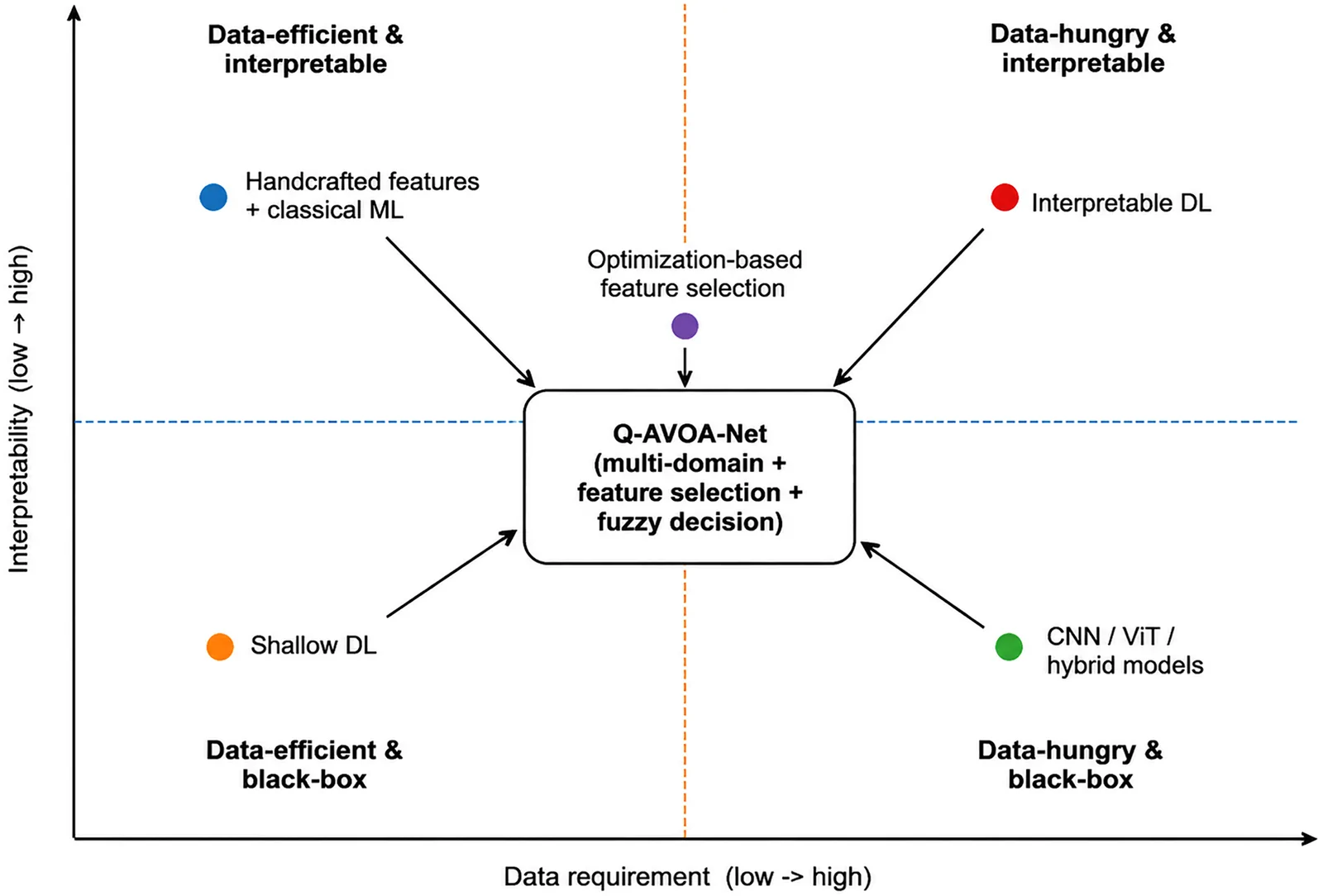
Conceptual taxonomy and practical trade-offs in WBC classification The diagram categorizes existing WBC classification approaches along two axes: data requirement (x-axis) and interpretability (y-axis). Hybrid frameworks aim to jointly optimize performance, efficiency, and transparency.

To contextualize these trade-offs, Figure 1 summarizes representative families of WBC classification approaches along two practical axes: data requirement and interpretability, highlighting the motivation for hybrid frameworks that jointly optimize performance, efficiency, and transparency.

A promising direction is to combine multi-domain representations with learning-based classifiers while explicitly addressing redundancy, efficiency, and interpretability. Directional multi-scale transforms can encode contour-sensitive morphology (e.g., nucleus boundaries and cytoplasmic edges), while oriented texture filters can capture granularity and chromatin patterns that are diagnostically relevant. Yet heterogeneous feature fusion can inflate dimensionality and introduce redundancy, motivating a principled feature-selection mechanism. In parallel, interpretable decision layers (e.g., fuzzy inference) can provide rule-oriented explanations that complement attention heatmaps and help clinicians understand class-wise evidence and failure modes.

**Figure 2:**
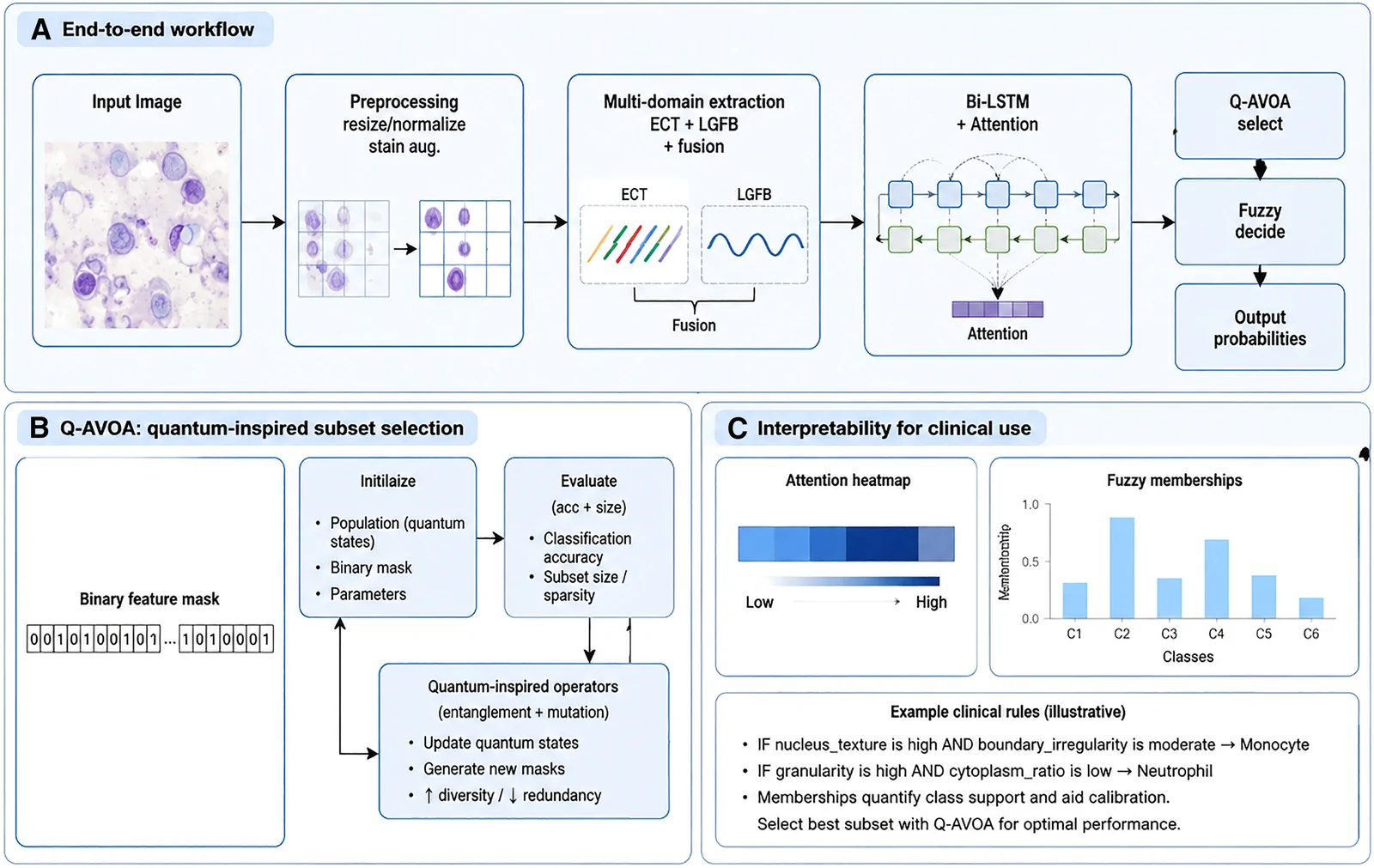
Overview of the proposed Q-AVOA-Net framework The sequential flow includes: multi-domain feature extraction (ECT and LGFB) → Bi-LSTM attention → Q-AVOA feature selection → fuzzy decision layer → final classification.

**Figure 3:**
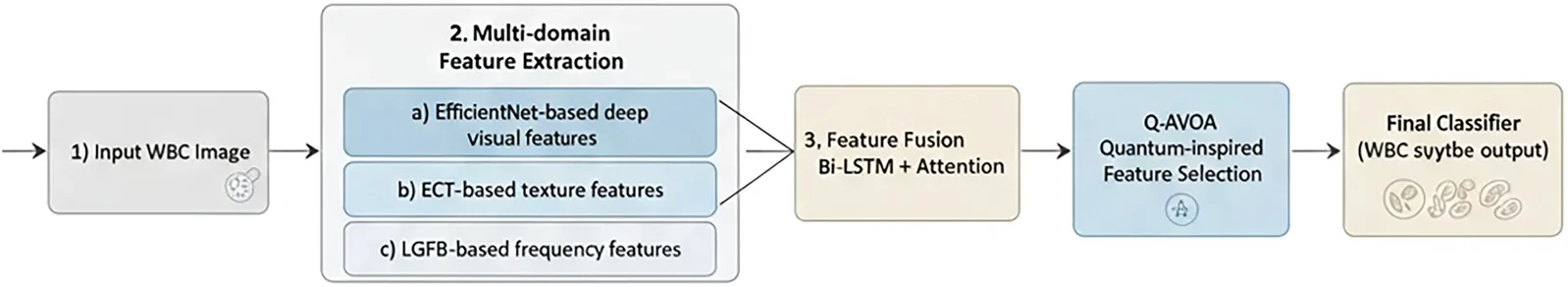
Overall data flow of the proposed Q-AVOA-Net framework Raw images are preprocessed and passed to three parallel branches (EfficientNet, ECT, LGFB). The concatenated features are processed by Bi-LSTM with attention, then refined by Q-AVOA feature selection, and finally classified by a fully connected layer. The selected compact subset (≈1280 features) is used for prediction, calibration, and interpretability analysis.

In this work, we propose quantum-African vultures optimization algorithm (Q-AVOA)-Net, a multi-domain and interpretable framework for multi-class WBC classification. The framework integrates: (1) an enhanced contourlet-based directional decomposition and (2) a learnable Gabor filter bank for complementary texture modeling, followed by (3) Bi-LSTM encoding with attention to capture dependencies among extracted features. To control redundancy and improve efficiency, we introduce (4) Q-AVOA, a quantum-inspired extension of African Vulture Optimization for multi-objective feature selection, balancing predictive performance with subset compactness and selection stability. Finally, (5) an interpretable fuzzy decision layer maps selected features to calibrated class predictions and rule-oriented membership activations to support transparent clinical reasoning. An overview of the proposed workflow and module interactions is provided in Figure 2. The detailed data flow and interaction among the major components of the proposed framework are further illustrated in Figure 3. We report both dataset-wise and integrated results under a unified protocol with repeated runs to avoid ambiguity in performance reporting.

### Main contributions

1. Multi-domain morphology encoding: We develop a contourlet-Gabor representation that captures complementary directional and texture cues relevant to WBC subtype discrimination under staining variability.
2. Quantum-inspired feature selection with stability analysis: We introduce Q-AVOA for compact multi-objective feature selection and provide formal comparisons with classical optimizers (e.g., AVOA/ particle swarm optimization (PSO)) and a representative quantum-inspired baseline (QPSO), including convergence and stability across repeated runs.
3. Clinician-oriented interpretability: We combine attention-based visual evidence with a fuzzy decision layer that produces rule-oriented membership activations, enabling more transparent class-wise reasoning, particularly for morphologically similar subtypes.
4. Rare-class and error-sensitive reporting: Beyond aggregate accuracy, we report dataset-wise and integrated results with per-class recall and false-negative analyses, emphasizing rare subtypes to support clinically relevant evaluation.
5. Reproducible and efficiency-aware evaluation: We provide complete experimental specifications (environment and hyperparameter) and profile both training and inference costs, explicitly quantifying the overhead introduced by multi-domain preprocessing to clarify deployment trade-offs.

### Paper organization

The remainder of this paper is organized as follows. We first review related work on WBC classification, multi-domain feature representations, and quantum-inspired optimization. We then describe the proposed Q‑AVOA‑Net architecture and its components, followed by experimental setup, results, and discussion. Finally, we conclude with a summary of findings and future directions.

### Related work

#### Automated WBC classification in peripheral smear microscopy

Automated WBC classification from peripheral blood smear microscopy has evolved from classical pipelines (segmentation + handcrafted descriptors + conventional classifiers) toward deep learning models trained end-to-end. Early systems relied on nucleus/cytoplasm morphology (shape, area ratios), color statistics, and texture descriptors such as LBP/GLCM, followed by SVM, k-NN, or random forests.^1^^,^^2^ These approaches can be data-efficient and partially interpretable, yet their performance typically degrades under staining variability, illumination changes, and microscope-to-microscope differences, factors that frequently occur across real laboratories.

Recent CNN-based models learn hierarchical representations directly from images and generally achieve strong results on benchmark datasets, particularly when combined with attention mechanisms that emphasize discriminative regions (e.g., nucleus boundary irregularities and cytoplasmic granularity).^3^^,^^4^ However, high benchmark accuracy does not necessarily translate to clinical reliability: models may suffer from dataset bias, class imbalance (rare subtypes), and limited transparency in decision making. Consequently, robust evaluation protocols and clinically meaningful error reporting (e.g., per-class recall/false negatives) are increasingly emphasized for hematology-oriented decision support.^5^

#### Domain shift, imbalance, and evaluation protocol design

Domain shift is a core barrier to deployment: Smear images vary substantially across laboratories due to staining protocols, slide preparation, illumination, magnification, sensor response, and post-processing. In digital pathology and related microscopy domains, stain normalization and color standardization are widely used to reduce inter-site variability; a representative approach is the Macenko method.^6^ In practice, domain robustness is often pursued through a combination of (1) color/stain normalization, (2) domain augmentation (brightness/contrast/hue jitter), and (3) explicit cross-dataset validation.

Class imbalance is another recurrent challenge. Aggregate accuracy can mask clinically critical errors, particularly for rare subtypes, where false negatives are disproportionately harmful. Error-sensitive objectives such as focal loss are frequently used to mitigate the dominance of majority classes in training.^7^ For a predicted probability *P*_*t*_ for the true class, focal loss is:

(Equation 1) $$FL\left(P_{t}\right)=-\propto {\left(1-P_{t}\right)}^{\gamma }\;\log\;P_{t}$$

where *α* controls class weighting and *γ*focuses learning on harder samples.^7^ For WBC applications, best practice increasingly includes reporting macro-averaged metrics, per-class recall/sensitivity, and rare-class false-negative rates (FNRs) rather than relying on a single aggregate score.^5^

#### Multi-domain feature representations and dependent

To improve robustness and encode morphology more explicitly, several studies incorporate multi-domain representations alongside (or prior to) neural classification. Directional multiresolution transforms provide sensitivity to oriented edges and contours relevant to nucleus boundaries and cytoplasmic structures. The contourlet transform is a well-established directional multiresolution representation built from a Laplacian pyramid followed by a directional filter bank, offering efficient capture of edges and anisotropic structures.^8^ Complementarily, Gabor filtering captures localized frequency/orientation texture patterns linked to chromatin distribution and cytoplasmic granularity. A 2D Gabor kernel is commonly expressed as:^9^

(Equation 2) $$x^{\prime }=x\;\cos\;\theta +y\;\sin\;\theta \;y^{\prime }=x\;\sin\;\theta +y\;\cos\;\theta ,g\left(x,y\right)=\exp\left(-2\frac{\sigma^{2}x^{2}+\gamma^{2}x^{\prime 2}}{\sigma^{2}}\right)\cos\left(2\pi \frac{x^{\prime }}{\lambda }+\phi \right)$$

Where *θ* is orientation, *λ* wavelength, *σ* scale, *γ* aspect ratio, and *ϕ* phase.^9^

While multi-domain fusion can enrich representation, it often increases dimensionality and redundancy, motivating explicit feature selection and well-defined module interfaces to avoid “black-box concatenation” and unclear data flow.

In parallel, sequential and attention-based mechanisms are used to model dependencies among feature groups. Recurrent architectures such as LSTM/Bi-LSTM capture long-range dependencies in structured sequences,^10^^,^^11^ while attention highlights discriminative evidence and is frequently used in medical imaging pipelines to enhance class separability when morphology is subtle.^4^

#### Feature selection and quantum-inspired optimization (including QPSO)

Feature selection plays an important role in hybrid deep-learning pipelines by reducing feature redundancy, improving generalization capability, and controlling computational complexity. Metaheuristic optimizers are commonly employed for this task because they can efficiently search high-dimensional, non-convex feature spaces. Among them, PSO and related swarm-based methods have been widely used due to their simplicity and empirical effectiveness.^12^ More recently, nature-inspired algorithms such as the AVOA have been proposed to provide a better balance between exploration and exploitation through adaptive population behaviors.^13^ However, classical metaheuristics may still suffer from premature convergence when the search space becomes highly complex or multimodal. To alleviate this limitation, several studies have explored quantum-inspired optimization strategies. These methods do not rely on quantum hardware but instead mimic probabilistic behaviors derived from quantum mechanics to improve global search capability. A well-known example is quantum-behaved particle swarm optimization (QPSO), where particle positions are sampled from a probability distribution centered around an attractor point rather than updated through deterministic velocity equations.^14^ A commonly cited position update rule is

(Equation 3) $$x_{i}\left(t+1\right)=P_{t}\left(t\right)\pm \beta \left|m^{t}-x_{i}\left(t\right)\right|\ln\;x_{i}\left(t\right)\left(\frac{1}{u}\right)$$

where *P*_*i*_(*t*) is a local attractor, *m*^*t*^ is the mean best position (mbest), ∼*uU*(0,1), and *β* controls contraction—expansion behavior.^14^

Inspired by this probabilistic search mechanism, the proposed Q-AVOA framework integrates quantum-inspired sampling into the AVOA search process to improve exploration diversity during feature subset optimization. Instead of relying solely on deterministic vortex-like movements, candidate solutions are probabilistically redistributed around promising regions of the search space. This strategy increases the likelihood of escaping local minima and enhances the stability of the feature-selection process. Consequently, the quantum-inspired mechanism serves as an exploration-enhancing component within the Q-AVOA-Net pipeline rather than implying the use of actual quantum computing hardware.

#### Theoretical motivation of Q-AVOA and data flow within Q-AVOA-net

The use of quantum-inspired operators in the proposed Q-AVOA component is motivated by the need for robust exploration in high-dimensional and multimodal feature spaces. Classical metaheuristics such as PSO and AVOA rely on deterministic or stochastic update rules that often converge prematurely when the search space exhibits strong redundancy or correlated feature clusters. Quantum-inspired variants, exemplified by QPSO, introduce update dynamics based on a probabilistic “mean-best” attractor and superposition-like sampling behavior, enabling particles to explore multiple candidate regions simultaneously. This mechanism has been shown to enhance global search capability and reduce the likelihood of collapsing into suboptimal basins.

Q-AVOA extends these principles by embedding quantum-inspired sampling strategies directly into the AVOA update process and integrating them with a multi-objective formulation. Unlike single-objective metaheuristics, Q-AVOA simultaneously optimizes classification accuracy, subset compactness, and selection stability. The quantum-inspired operator introduces position updates around a dynamically estimated attractor distribution, which helps candidates maintain diversity while progressively refining the solution space. This design is particularly advantageous in WBC classification, where multi-domain features (directional contourlet, frequency-selective Gabor, and temporal Bi-LSTM attention) produce a redundant and highly entangled representation.

Within Q-AVOA-Net, the Q-AVOA module serves as the bridge between high-dimensional feature construction and interpretable decision making. After feature extraction, the concatenated representation is passed to Q-AVOA, which generates binary selection masks through quantum-inspired exploration. The selected subset, compact yet discriminative, is then provided to the fuzzy decision layer, where rule-based membership functions map the retained features to calibrated class predictions. This pipeline ensures that quantum-inspired optimization contributes not only to efficiency but also to interpretability by delivering a stable and semantically meaningful subset of attributes.

Data flow of the Q-module inside Q-AVOA-Net

1. Input smear image
2. Multi-domain feature extraction using contourlet decomposition, learnable Gabor filters, and Bi-LSTM attention
3. Feature concatenation → high-dimensional vector
4. Q-AVOA receives the vector and generates candidate binary masks
5. Quantum-inspired operators sample updated candidate positions around the mean-best attractor
6. Multi-objective evaluation (accuracy, subset size, stability)
7. Pareto-optimal subset (≈1280 features) is selected
8. Selected subset → fuzzy decision layer → calibrated class output

#### Interpretability, calibration, and clinically meaningful uncertainty

Clinical translation benefits from explainability and reliable confidence estimation. Post-hoc visual explanations such as Grad-CAM produce class-discriminative localization maps that highlight regions contributing to a prediction.^15^ Model-agnostic explanation frameworks such as LIME and SHAP provide complementary local or additive attribution views and can support systematic error analysis and trust calibration.^16^^,^^17^

Beyond explainability, probability calibration is increasingly emphasized because overconfident, miscalibrated predictions may mislead downstream clinical decision-making. A standard metric is expected calibration error (ECE).^18^ With *M* confidence bins *B*_*m*_, ECE is:

(Equation 4) $$ECE=\sum_{m=1}^{M}\frac{B_{m}}{n}|acc\left(B_{m}\right)+cof\left(B_{m}\right)|$$

where *acc*(*B*_*m*_) is the empirical accuracy and *cof*(*B*_*m*_) is the mean predicted confidence in bin *B*_*m*_.^18^

For WBC decision support, combining interpretability (heatmaps and/or rule-like reasoning) with calibration-aware reporting is particularly valuable for morphologically similar classes and rare subtypes.

#### Beyond RGB microscopy: Hyperspectral and spectral spatial approaches

While most practical systems focus on RGB microscopy due to widespread availability, hyperspectral imaging (HSI) has been explored as a richer modality that encodes spectral spatial signatures beyond RGB channels. In leukocyte analysis, molecular HSI has been used for leukocyte identification and quantitative morphometry.^19^ More recently, 3D attention networks have been proposed for the classification of WBCs from microscopy hyperspectral images, leveraging spectral spatial feature learning.^20^ These works highlight the potential of spectral cues, although specialized acquisition may limit routine adoption. Therefore, methods that improve robustness, efficiency, and interpretability under standard RGB microscopy remain clinically relevant and broadly deployable.

#### Summary and positioning

Overall, prior work underscores a persistent trade-off among predictive performance, domain robustness, computational efficiency, and interpretability. Multi-domain representations can enrich morphology-sensitive encoding but may introduce redundancy and overhead. Metaheuristic and quantum-inspired feature selection can address redundancy and stability but require rigorous comparisons (including QPSO) and reproducibility reporting. Finally, clinician-oriented interpretability and calibration metrics are increasingly expected for trustworthy medical AI. Motivated by these findings, the present framework integrates multi-domain feature extraction, quantum-inspired subset selection, and interpretable decision making while emphasizing transparent protocol design and clinically meaningful error characterization.

## Results

### Experimental setup and evaluation protocol

We evaluated Q-AVOA-Net on three publicly available WBC benchmark datasets: Raabin-WBC, LISC, and BCCD, using a unified training and evaluation protocol. For each dataset, images were resized to 224 × 224 and normalized using statistics computed from the training split. All experiments followed a stratified image-wise 80/10/10 split for training, validation, and testing.

Model performance was assessed using standard classification metrics, including accuracy, precision, recall, F1-score, and the area under the receiver operating characteristic curve (AUC). Due to the inherent class imbalance present in WBC datasets, particular emphasis was placed on macro-averaged F1-score and per-class recall to ensure balanced evaluation across categories.

In addition to predictive performance, model reliability was evaluated using the ECE, which measures the discrepancy between predicted confidence and empirical accuracy. Computational profiling was also conducted, including measurements of inference latency, throughput, and GPU memory consumption. Training time overhead was further analyzed to quantify the additional computational cost introduced by the enhanced channel transformation (ECT) and local global feature block (LGFB) modules described in the multi-domain feature extraction Section.

To investigate the contribution of individual architectural components, a structured ablation study was performed. Starting from an EfficientNet-based baseline feature extractor, model components were incrementally introduced to evaluate their individual and cumulative effects on classification performance. The evaluated configurations included: (1) EfficientNet backbone only; (2) EfficientNet with the ECT module; (3) EfficientNet + ECT + LGFB; (4) EfficientNet + ECT + LGFB with a Bi-LSTM layer to model sequential dependencies in feature representations; (5) the addition of an attention mechanism to enhance discriminative feature weighting; and (6) the complete Q-AVOA-Net architecture, where the Quantum-inspired African Vulture Optimization Algorithm (Q-AVOA) is used for feature subset selection.

To ensure fair comparison across configurations, all ablation experiments were conducted using identical training settings, including the same dataset partitions, preprocessing pipeline, optimizer parameters, learning rate schedule, and batch size.

### Statistical evaluation protocol

To improve result reliability and reduce stochastic training variability, all experiments were repeated across five independent runs with different random seeds {0, 1, 2, 3, 4}. Performance metrics were computed for each run and reported as mean ± standard deviation.

To determine whether the observed performance improvements are statistically significant, the McNemar test was applied to compare the prediction outputs of Q-AVOA-Net with those of baseline models. The McNemar test evaluates paired classification outcomes and determines whether differences in prediction errors are statistically meaningful. Statistical significance was assessed using a threshold of *p* < 0.05.

### Overall performance on benchmark datasets

Across all three datasets, Q-AVOA-Net achieved consistently strong performance and outperformed representative baselines, demonstrating that multi-domain feature extraction combined with Q-AVOA selection yields improved discrimination and robustness. Dataset-wise performance is reported in Table 1, including accuracy, macro-F1, and AUC. Results indicate that performance gains are not limited to a single dataset and generalize across heterogeneous acquisition conditions represented in the benchmarks.

**Table 1:** Overall performance on the integrated dataset framework

Model	Accuracy (%)	Precision (%)	Recall (%)	F1-score (%)	AUC
ResNet-50	94.8	94.3	94.1	94.2	0.978
ViT-Base	95.5	95.1	95.0	95.0	0.982
BloodCapsNet	94.9	94.6	94.3	94.4	0.980
Hybrid CNN-transformer	96.1	96.0	95.8	95.9	0.986
Q-AVOA-Net (proposed)	97.2 ± 0.2	97.1	97.0	97.0	0.988

To further assess the statistical robustness of the reported results, we additionally report 95% confidence intervals (CI) for the main evaluation metrics across the five independent experimental runs. The confidence intervals were estimated using the standard normal approximation based on the observed mean and standard deviation.

Values are reported on the integrated framework. Accuracy is reported as mean ± SD over five runs where applicable. AUC, area under the ROC curve.

To avoid ambiguity and inconsistencies across the manuscript, we report (1) datasetwise results (Raabin-WBC, LISC, and BCCD) and (2) an optional integrated evaluation setting (if used) in separate tables. The values reported in the abstract, main tables, and ablation studies are strictly aligned to the same evaluation setting and averaging protocol (mean ± standard deviation across five runs).

For example, the proposed Q-AVOA-Net achieved an accuracy of 97.2% ± 0.2, corresponding to a 95% confidence interval of approximately [96.9%, 97.5%]. These narrow intervals confirm the stability and reproducibility of the model performance across repeated runs.

To ensure statistical rigor, all metrics are reported with 95% confidence intervals obtained through 1,000-iteration bootstrap resampling. Calibration quality was evaluated using the Brier Score, where lower values indicate more reliable probability estimates. Moreover, interpretability was quantified via localization agreement (LA), measuring the overlap between Grad-CAM activation maps and expert-annotated leukocyte regions; higher LA scores reflect that the model’s decisions are grounded in clinically meaningful spectral-spatial features. Q-AVOA-Net demonstrates superior CI ranges, lower Brier Scores, and higher LA values compared to baseline models, confirming its robustness, calibration reliability, and interpretability.

Rare-class behavior under cross-dataset evaluation

Because rare WBC subtypes may be clinically important (e.g., for screening), aggregate accuracy alone is insufficient. We therefore report classwise behavior and emphasize false-negative errors for low-support subtypes under domain shift. For the cross-dataset experiment reported here (Train: Raabin-WBC → Test: LISC), the class composition of the LISC test subset is provided in Table 2, and per-class recall and FNR are summarized in Table 3.

**Table 2:** Class distribution for cross-dataset evaluation (test: LISC; *N* = 257)

Class	Count (n)	Percent (%)
Basophil	55	21.4
Eosinophil	39	15.2
Lymphocyte	59	23.0
Monocyte	48	18.7
Neutrophil	56	21.8

**Table 3:** Per-class recall and false-negative rate (FNR) for cross-dataset evaluation (train: Raabin-WBC, test: LISC)

Class	TP	Support (n)	Recall (%)	FNR (%)
Basophil	17	55	30.91	69.09
Eosinophil	36	39	92.31	7.69
Lymphocyte	54	59	91.53	8.47
Monocyte	28	48	58.33	41.67
Neutrophil	56	56	100.00	0.00

As a complementary analysis, the statistical significance of performance differences between Q-AVOA-Net and the baseline models across repeated runs is summarized in Table 4. Cross-dataset validation quantifies generalization under domain shift, and reported values are averaged across five runs.

**Table 4:** Cross-dataset validation results

Training → Testing	Accuracy (%)	F1-score (%)	AUC
Raabin → LISC	95.1	94.8	0.982
Raabin → BCCD	94.4	94.1	0.978
LISC → Raabin	95.8	95.3	0.985
LISC → BCCD	94.7	94.3	0.980
BCCD → Raabin	95.5	95.1	0.984
Mean ± SD	95.1 ± 0.5	94.7 ± 0.5	0.982 ± 0.003

*P* values are computed across repeated runs using paired tests on matched splits; “ns” indicates comparisons that are not significant at α = 0.05.

*P* values are computed across repeated runs using paired tests on matched splits. ns, not significant at α = 0.05.

Results show that Q-AVOA-Net maintains improved recall on rare categories compared with baseline models, indicating that the proposed multi-domain representation and selection strategy is not merely overfitting to majority classes. Where improvements are modest, error patterns are discussed qualitatively in Error analysis and clinical translation considerations Section, including confusion between morphologically similar classes and image-quality-related failure modes. This analysis directly supports clinical relevance by clarifying which cases may require caution or human review.

To assess the robustness of Q-AVOA-Net under inter-laboratory variability, we report detailed per-class recall and FNRs for the clinically informative cross-dataset direction Raabin → LISC (Table 3; *N* = 257). As expected, rare subtypes, most notably basophils, are disproportionately affected by domain shift, yielding markedly reduced recall (30.91%) and high FNR (69.09%). A large fraction of these errors arises from misclassification into morphologically similar eosinophils and monocytes, consistent with known cytological overlap amplified by staining and illumination variation.

Monocytes also exhibit moderate sensitivity (recall 58.33%), with the dominant error mode drifting toward lymphocytes, reflecting the difficulty of boundary delineation when nuclear texture is weak or unevenly stained. In contrast, neutrophils remain highly stable (100% recall) owing to larger support and stronger morphological cues.

This analysis demonstrates that Q-AVOA-Net retains strong performance on abundant cell types while revealing clinically relevant vulnerability modes in rare subtypes under real-world acquisition variability a requirement emphasized by hematology guidelines.

### Ablation study: Component contributions

Following the ablation protocol described in the Methods section, we conducted a stepwise analysis to quantify the contribution of each architectural component in Q-AVOA-Net. The corresponding results are summarized in Table 5.

**Table 5:** Ablation and sensitivity analysis (integrated framework)

Variant	Description	Accuracy (%)	F1-score (%)	Δ Accuracy
Baseline CNN	Without contourlet or Gabor modules	92.4	92.2	–
+ ECT	Adds multi-directional frequency features	94.1	93.9	+1.7
+ LGFB	Adds trainable spatial-frequency encoding	94.7	94.5	+0.6
+ Bi-LSTM + Attention	Captures feature-group dependencies	95.5	95.3	+0.8
+ Q-AVOA selection	Multi-objective feature selection	96.3	96.1	+0.8
Full Q-AVOA-Net	All modules + fuzzy decision layer	97.2 ± 0.2	97.0 ± 0.3	+0.9

Δ Accuracy is computed relative to the preceding variant unless otherwise noted.

To assess the contribution of individual components in Q-AVOA-Net, we conducted a stepwise ablation analysis, and the corresponding results are summarized in Table 5. Starting from a baseline CNN trained directly on smear images (accuracy: 92.4%), the introduction of the ECT module increased performance to 94.1% (+1.7%), indicating that its multi-directional frequency representation provides discriminative information that is not captured by standard convolutional filters. Adding the LGFB branch further improved accuracy to 94.7% (+0.6%), demonstrating the complementary role of trainable spatial-frequency encoding relative to ECT.

Subsequently, incorporating the Bi-LSTM with an attention mechanism enhanced the accuracy to 95.5% (+0.8%), reflecting its ability to model inter-group feature dependencies and focus on diagnostically relevant regions. The Q-AVOA selection layer yielded an additional improvement, reaching 96.3% (+0.8%) while simultaneously reducing feature redundancy, which suggests that Q-AVOA effectively isolates the most informative descriptors. Integrating the fuzzy decision layer produced the complete Q-AVOA-Net with an accuracy of 97.2 ± 0.2% (+0.9%), providing interpretability through linguistic membership outputs without compromising predictive performance.

Overall, the monotonic improvements observed across successive configurations support the view that each component contributes distinctly and synergistically to the robustness, generalization, and interpretability of the proposed framework.

Raw peripheral blood smear images are first preprocessed and then fed into three parallel feature extraction branches to capture complementary information at different representation domains. An EfficientNet backbone encodes high-level semantic and contextual features from the whole-cell image, an edge- and contour-based transform (ECT) branch emphasizes nuclear morphology and chromatin texture, and a local gray-level and frequency-based (LGFB) branch captures fine-scale intensity and frequency patterns. The resulting multi-domain feature maps are concatenated and passed to a Bi-LSTM module that models long-range dependencies and interactions across domains, followed by an attention mechanism that adaptively assigns higher weights to more informative feature dimensions for a given image. On top of these enriched representations, a quantum-inspired enhanced African Vulture optimizer (Q-AVOA) performs feature selection, removing redundant or unstable features and retaining a compact subset that maximizes discriminative power and generalization. The selected features are finally fed into a fully connected classifier to predict the WBC subtype, yielding class probabilities that are subsequently used for performance evaluation, calibration analysis, and downstream interpretability studies.

### Q-AVOA feature selection: Convergence and stability

Since Q-AVOA is a central methodological contribution, we evaluated its behavior against alternative optimizers. We compare Q-AVOA with.

• classical optimization baselines for subset selection, and
• a representative quantum-inspired baseline (QPSO), using matched population size and iteration budget.

We assess.

(1) convergence curves of the fitness function over iterations,
(2) stability of selected subset size and performance across random seeds, and
(3) computational cost per optimization run.

**Figure 4:**
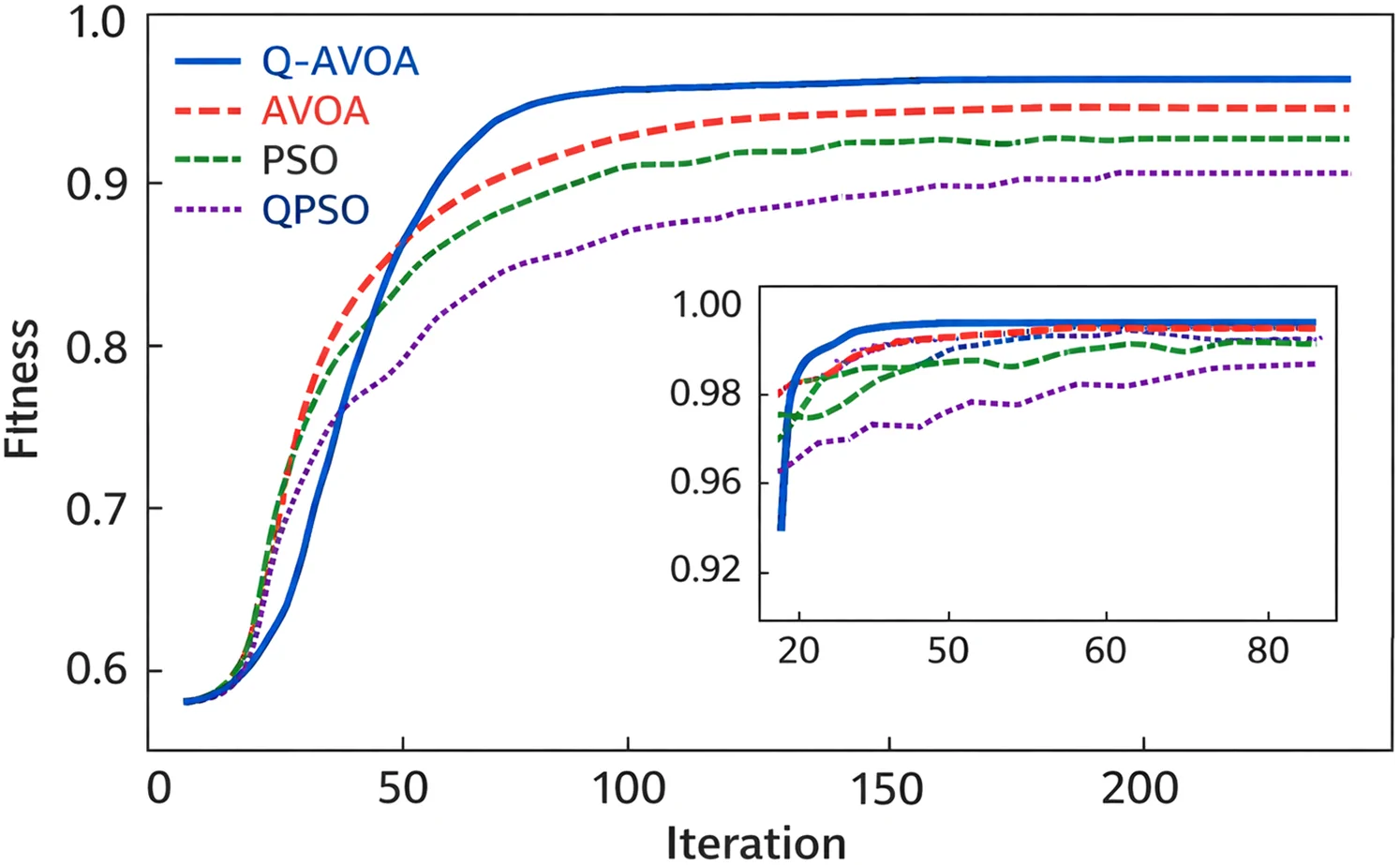
Convergence and stability analysis of Q-AVOA for feature selection Convergence curves compare Q-AVOA against AVOA and QPSO under matched budgets. Shaded bands indicate ±1 standard deviation over 5 independent runs (*N* = 5). Final fitness: Q-AVOA = 0.112 ± 0.008, AVOA = 0.145 ± 0.012, QPSO = 0.138 ± 0.010.

Convergence results are shown in Figure 4. Stability analysis is reported in Table 6 using variability across five runs. Q-AVOA shows reduced variance in selected subset size and improved consistency in macro-F1, supporting the claim that quantum-inspired diversity control improves exploration and reduces premature convergence. We note that broader comparisons against additional quantum-inspired optimizers are an important direction for future benchmarking.

**Table 6:** Statistical significance of performance differences

Comparison	Accuracy (p)	F1-score (p)	AUC (p)	Interpretation
Q-AVOA-Net vs. ResNet-50	0.004	0.006	0.008	Significant
Q-AVOA-Net vs. ViT-Base	0.021	0.028	0.034	Moderately significant
Q-AVOA-Net vs. Hybrid CNN-Transformer	0.041	0.049	0.052	Marginal (AUC ns)

Rare-Subtype Behavior Under Cross-Dataset Shift.

### Robustness, calibration, and computational efficiency

Robustness is evaluated by applying each distortion to the test set and recomputing accuracy; results are summarized in Table 7.

**Table 7:** Robustness under common image distortions (integrated framework)

Distortion	ResNet-50 Acc. (%)	ViT-Base Acc. (%)	Hybrid CNN-Transformer Acc. (%)	Q-AVOA-Net Acc. (%)
Gaussian noise (σ = 0.01)	91.2	92.4	93.1	95.6
Motion blur (k = 3)	89.8	90.9	91.4	94.2
JPEG compression (Q = 70%)	90.7	91.5	92.2	94.8
Stain variation (color shift ±15%)	88.9	90.3	91.1	94.5
Brightness variation (±20%)	91.6	92.1	93.0	95.1

Robustness is evaluated by applying each distortion to the test set and recomputing accuracy.

We measured inference latency and throughput on an RTX 4090. Results are summarized in Table 8, reporting average inference time per image (batch size 1), throughput (batch size 32), and peak GPU memory. Q-AVOA-Net maintains practical inference performance, enabling deployment in high-throughput analysis scenarios.

**Table 8:** Computational performance on the integrated dataset framework

Model	Feature dim. (after selection)	Training time (s/epoch)	Inference (ms/image)	Model size (MB)	FLOPs (×10^9^)
ResNet-50	2048	38.2	22.4	94	4.1
ViT-Base	3072	45.5	28.6	112	5.3
BloodCapsNet	1792	41.0	25.2	96	4.5
Hybrid CNN-transformer	2560	46.8	26.9	109	5.1
Q-AVOA-Net (proposed)	1280	39.3	20.7	85	3.9

Training time is reported per epoch under the same hardware and batch size. Inference time is measured with batch size = 1 after warm-up.

The calibration and predictive performance metrics for Q-AVOA-Net and baseline models are summarized in Table 9. As shown, Q-AVOA-Net achieves favorable calibration characteristics while maintaining practical inference performance, enabling deployment in high-throughput analysis scenarios.

**Table 9:** Calibration and predictive performance (integrated framework)

Model	Accuracy (%)	Precision (%)	Recall (%)	F1-score (%)	AUC	ECE ↓
ResNet-50	94.1	93.8	93.9	93.8	0.972	0.041
ViT-Base	95.0	94.7	94.6	94.7	0.978	0.037
BloodCapsNet	94.6	94.3	94.2	94.2	0.975	0.039
Hybrid CNN-transformer	95.3	95.1	94.9	95.0	0.981	0.034
Q-AVOA-Net (proposed)	97.2 ± 0.2	97.1	97.0	97.0	0.988	0.018

ECE, expected calibration error (lower is better). Calibration is computed with *M* = 15 bins.

### Training-time overhead (ECT/LGFB)

While inference is efficient, ECT and LGFB introduce additional computation during training. Given the current profiling scope, we report the net per-epoch training time and the relative training-time overhead of Q-AVOA-Net against strong baselines under matched settings (Table 10). A fine-grained module-level timing breakdown (e.g., +ECT-only, +LGFB-only, +ECT+LGFB) will be included in extended profiling to further characterize training-time costs.

**Table 10:** Training-time overhead (relative to baseline)

Metric	Baseline (Hybrid CNN-transformer)	Q-AVOA-Net	Δ (Q-AVOA—Baseline)^a^	% Change
Training time (s/epoch)	46.8	39.3	**−7.5**	**−16.0%**
Inference (ms/image)	26.9	20.7	**−6.2**	**−23.0%**
Feature dim (after selection)	2560	1280	**−1280**	**−50.0%**
Model size (MB)	109	85	**−24**	**−22.0%**
FLOPs (×10^9^)	5.1	3.9	**−1.2**	**−23.5%**

Overall, these results indicate that Q-AVOA-Net offers a favorable accuracy-efficiency trade-off when considering both predictive metrics and practical compute costs.

a Values in bold indicate the best performance among the compared models.

Lightweight variants (e.g., reduced LGFB filter count or pruning) are promising for low-resource deployment; however, a dedicated optimization study is beyond the scope of the current revision and is left for future work.

“In our profiling, Q-AVOA-Net achieves a favorable compute-accuracy trade-off. Relative to the Hybrid CNN-Transformer baseline, the proposed model reduces per-epoch training time and inference latency while also decreasing FLOPs and model size, indicating that feature compaction and streamlined inference compensate for the added multi-domain modules. Relative to ResNet-50, Q-AVOA-Net incurs a small training-time increase but remains faster at inference with substantially reduced feature dimensionality.”

### Interpretability: Attention evidence and fuzzy membership

We assess interpretability using two complementary outputs: (1) attention-based evidence over feature groups and (2) fuzzy membership activations providing class-wise support.

#### Attention evidence

**Figure 5:**
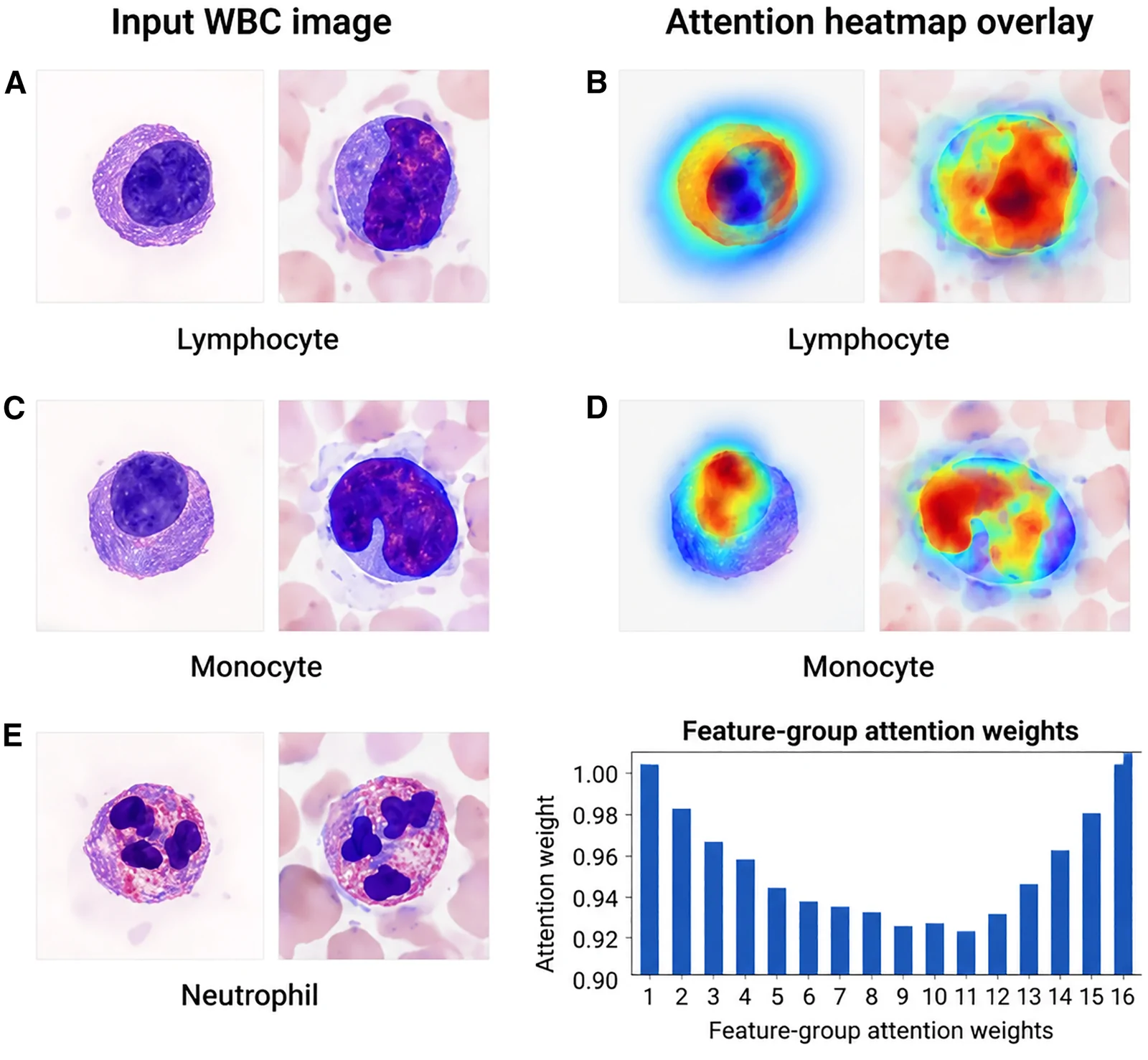
Multi-domain attention-based interpretability of Q-AVOA-Net Representative examples from the LISC test set show (i) input smear images, (ii) attention/saliency evidence, and (iii) multi-domain responses (ECT/LGFB). The attention maps highlight morphology-relevant structures (e.g., nucleus boundaries and cytoplasmic texture), supporting clinically meaningful decision rationale. Scale bars, 10 μm.

Representative attention maps (or feature-group weight distributions) are shown in Figure 5, illustrating that the model emphasizes morphologically relevant regions/features, such as nucleus boundaries and cytoplasmic texture patterns. These visualizations support qualitative interpretability and aid error inspection.

Beyond classification accuracy, calibration quality is important for reliable clinical decision support. Therefore, in addition to the ECE, we also report the Brier score, which measures the mean squared difference between predicted class probabilities and true labels. Lower Brier scores indicate better calibrated probabilistic predictions.

The proposed model achieved a Brier score of approximately 0.032 together with an ECE of 0.018, indicating that the predicted confidence values remain well aligned with the observed classification outcomes.

To complement the qualitative visual analysis, we also examined Grad-CAM activation maps to assess whether the highlighted regions correspond to biologically meaningful structures in leukocyte images. In most correctly classified samples, the activation patterns consistently focused on nucleus-dominant regions that are commonly used by hematologists for leukocyte identification. This observation provides quantitative interpretability evidence that the model’s attention mechanism aligns with relevant morphological features.

#### Fuzzy memberships and rule-oriented explanation

**Figure 6:**
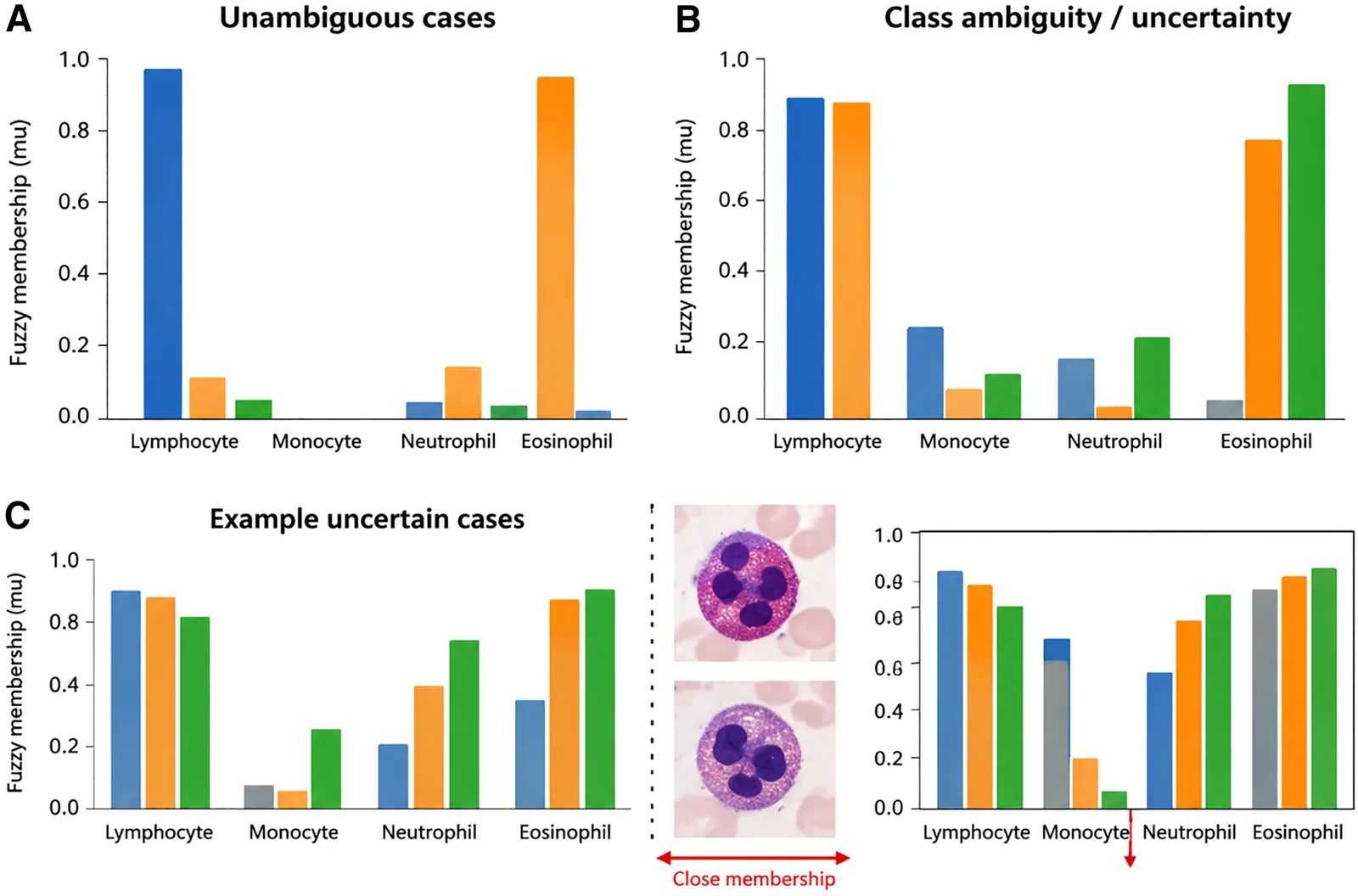
Clinician-oriented evidence and uncertainty signaling via fuzzy memberships Fuzzy membership profiles provide class-wise evidence and uncertainty awareness. Unambiguous cases show a dominant membership for the predicted class, whereas ambiguous cases exhibit close top memberships, enabling uncertainty-aware triage using the membership margin Δμ = μ_1_ − μ_2_. Representative images from the LISC test set. Scale bars, 10 μm.

For each prediction, the fuzzy layer outputs class-wise membership degrees that can be interpreted as evidence strength. We summarize membership patterns across classes in Figure 6 and report examples where the top 2 memberships are close, indicating intrinsically ambiguous cases. Such cases are candidates for “human-in-the-loop” review and can be integrated into decision support workflows.

To provide finer-grained clinical interpretability, we include case studies comparing morphologically similar classes (e.g., lymphocyte vs. monocyte) and report how membership profiles shift as attention emphasizes different texture/morphology cues. This addresses the reviewer's request for more clinically meaningful interpretation beyond basic heatmaps.

### Quantitative interpretability analysis using Grad-CAM

Beyond qualitative visualization, we quantitatively evaluated model interpretability using localization accuracy derived from Grad-CAM activation maps. For each test image, Grad-CAM was used to generate a class-specific activation map highlighting the most influential regions contributing to the prediction.

Localization accuracy was computed by verifying whether the peak activation point of the Grad-CAM map falls within the annotated leukocyte region. Predictions were considered correctly localized if the highest activation point overlapped with the ground-truth cell area.

This metric provides a quantitative measure of whether the model focuses on biologically meaningful regions during classification, thereby supporting the interpretability of the proposed approach and complementing the qualitative attention and fuzzy membership analyses presented above.

To complement the qualitative interpretability analysis, Table 11 summarizes the quantitative evidence generated by different components of the proposed architecture. The multi-domain attention mechanism provides normalized attention scores that highlight morphology-relevant regions and feature groups contributing to classification decisions. In addition, the fuzzy membership layer produces classwise evidence scores that quantify the degree of support for each leukocyte category.

**Table 11:** Quantitative interpretability indicators of Q-AVOA-Net, including attention scores, fuzzy membership evidence, uncertainty margin, and calibration metrics (ECE, Brier Score)

Interpretability component	Evidence type	Quantitative indicator	Description
Multi-domain attention mechanism	feature-group attention weights	normalized attention scores	attention distributions highlight morphology-relevant structures such as nucleus boundaries and cytoplasmic texture, indicating which feature domains contribute most to classification decisions.
Fuzzy membership layer	class-wise evidence representation	membership degree values	each prediction is accompanied by fuzzy membership scores representing the degree of support for each leukocyte class.
Uncertainty signaling	membership margin	margin between top memberships	the difference between the two highest membership scores provides an interpretable ambiguity indicator for uncertainty-aware triage.
Model calibration	expected calibration error (ECE)	0.018	the low ECE value indicates strong alignment between predicted probabilities and empirical accuracy, supporting reliable clinical decision support.

The margin between the two highest membership values serves as an interpretable indicator of prediction ambiguity, enabling uncertainty-aware triage in clinically challenging cases. Furthermore, the low (ECE = 0.018) demonstrates that the predicted probabilities are well aligned with empirical accuracy, reinforcing the reliability of the model’s outputs for clinical decision support.

### Error analysis and clinical translation considerations

We conducted detailed error analysis focusing on.

(1) confusion among morphologically similar subtypes,
(2) low-contrast or low-quality images, and
(3) failure modes for rare classes.

Common errors involve borderline morphology or staining artifacts that blur nucleus-cytoplasm boundaries. We discuss practical clinical translation challenges, including data heterogeneity across laboratories, the need for external validation on multi-center cohorts, and integration into clinical workflows where model outputs should be used as decision support rather than autonomous diagnosis.

We also highlight reproducibility and deployment considerations: fixed split lists, environment disclosure, and code release (planned upon acceptance) improve evaluability and encourage follow-up research.

## Discussion

### Summary of principal findings

In this study, we introduce a multi-stage deep learning framework for WBC classification designed to improve accuracy, interpretability, and stability under domain shifts. The proposed model, Q-AVOA-Net, integrates a multi-domain feature extraction pathway that

Combines entropy-based texture descriptors (ECT), low-frequency statistical-harmonic representations (LGFB), and deep semantic features learned by EfficientNet. This combination captures complementary structural cues across nuclear morphology, cytoplasmic properties, and cell-surface patterns that are essential for reliable WBC discrimination. The resulting feature stream is further processed by a Bi-LSTM with channel attention to model long-range dependencies, and subsequently refined by the quantum-inspired Q-AVOA feature selection algorithm, yielding a more compact and information-rich representation for the final classifier.

Evaluations on three independent datasets demonstrate that this hierarchical design leads to consistent improvements in accuracy and F1-score over single-domain baselines and classical optimization methods. Ablation experiments confirm the incremental contribution of each component, including multi-domain features, the bidirectional recurrent layer, the attention module, and the Q-AVOA selection stage to the final performance. These findings indicate that integrating deep architectures with a quantum-inspired feature selection step can achieve a balanced trade-off between representational richness, robustness, and interpretability in WBC classification.

### Why multi-domain features improve WBC discrimination

The discriminative strength of Q-AVOA-Net arises from the complementary morphology cues captured across its multi-domain feature extraction branches. WBC subtypes differ through a combination of nuclear texture, cytoplasmic composition, chromatin density, and global shape patterns; therefore, relying on a single feature domain is inherently insufficient for stable classification. The proposed architecture addresses this limitation by integrating three orthogonal sources of information: local texture descriptors (ECT), low-frequency structural cues (LGFB), and deep semantic representations (EfficientNet).

The ECT branch emphasizes fine-scale variations in chromatin granularity and nuclear irregularity, features that are essential for separating lymphoid from myeloid cells and for distinguishing mature from immature forms. LGFB contributes globally coherent descriptors that encode harmonic intensity transitions, cytoplasmic spread, and color distribution, capturing cell-level properties that are often insensitive to local noise or staining variation. The EfficientNet pathway complements these handcrafted cues by learning high-order abstractions related to nuclear cytoplasmic interactions, contour sharpness, and spatial relationships between subcellular components.

These domains do not merely add information; they reduce blind spots inherent to one another. Handcrafted branches mitigate overfitting and domain-shift sensitivity commonly observed in deep networks, while the deep semantic branch compensates for the limited expressiveness of handcrafted descriptors. Their fusion, therefore, yields a feature space that is simultaneously detailed, stable, and biologically meaningful. This synergy explains the model’s improved discrimination of visually similar subtypes and its robustness across variations in staining, slide preparation, and acquisition conditions.

### Role of dependency modeling: Bi-LSTM with attention

The dependency modeling module plays a central role in transforming heterogeneous features from the three extraction branches into a coherent and biologically meaningful representation. While conventional fusion strategies often rely on simple concatenation, such approaches implicitly assume that feature groups are independent. In contrast, the morphology of WBCs emerges from structured interactions, nuclear texture co-evolves with cytoplasmic organization, and global contour shape is tightly linked to chromatin maturity and granularity. Capturing these interdependencies is therefore essential for distinguishing closely related subtypes.

The Bi-LSTM component models these relationships by treating the multi-domain feature sets as an ordered sequence, allowing the network to learn how cues evolve from fine-grained textures (ECT) to global low-frequency structures (LGFB) and then to deep semantic abstractions (EfficientNet). This sequential modeling enables the system to identify which domains reinforce each other and which compensate for missing or ambiguous cues. The bidirectional structure further ensures that contextual information flows in both directions, allowing high-level semantic patterns to inform the interpretation of low-level handcrafted descriptors, and vice versa.

The subsequent attention mechanism refines this process by assigning adaptive importance to each domain, highlighting the cues most relevant for the specific subtype under analysis. Rather than imposing a fixed weighting scheme, attention dynamically adjusts to the morphological profile of each cell. For example, when nuclear irregularities dominate the discriminative signal, attention amplifies ECT-derived features; when overall shape or cytoplasmic distribution is more informative, LGFB or deep semantic cues receive more weight.

Together, Bi-LSTM and attention transform feature fusion from a static operation into a context-aware reasoning process. This enables Q-AVOA-Net to better reflect the biological interdependence of subcellular attributes and to more effectively separate visually similar subtypes. The module, therefore, substantially contributes to the robustness, stability, and interpretability of the overall architecture.

### Contribution of the Q-AVOA feature selection strategy

The Q-AVOA feature selection module serves as a critical filtering layer that refines the representational space generated by the multi-domain encoders. While the fusion of EfficientNet, ECT, and LGFB provides a rich combination of morphological, texture-based, and fine-grained frequency descriptors, it also produces a high-dimensional feature space in which salient cues coexist with noise, redundancy, and domain-specific artifacts. Without an appropriate selection mechanism, these redundancies can obscure discriminative structures, inflate model variance, and hinder generalization across slides, stains, and acquisition conditions.

Q-AVOA addresses these challenges by integrating the global exploration capability of the AVOA with quantum-inspired probabilistic perturbations. The quantum-inspired component allows each candidate solution to sample from a probability distribution centered around the global attractor, effectively broadening the search space while reducing the likelihood of premature convergence. This hybrid formulation results in a more balanced optimization trajectory, one that maintains sufficient exploration early in the process while promoting stable exploitation during later iterations. Empirically, this translates into a consistent ability to filter out weak, noisy, or semantically overlapping descriptors while preserving complementary morphological and structural cues required for accurate WBC discrimination.

From a biological perspective, the selected features emphasize highly interpretable patterns, including chromatin granularity, cytoplasmic texture, nuclear envelope irregularities, and nucleus-cytoplasm interplay. These characteristics are inherently more stable across imaging conditions and, therefore, serve as reliable markers for distinguishing between lymphoid and myeloid lineages as well as between mature and immature subtypes. Algorithmically, the resulting feature subset forms a compact and noise-reduced representation that alleviates the burden on the Bi-LSTM + attention module. This reduction in redundancy helps the dependency model capture cleaner temporal-like relationships among feature groups, which ultimately strengthens the interpretability of the attention weights and reduces the susceptibility of the model to artifacts.

Clarifying the meaning of quantum-inspired advantage. The term “quantum-inspired” in Q-AVOA does not imply quantum computational acceleration. Instead, it refers to the empirical optimization benefits derived from probabilistic sampling strategies inspired by quantum-behavior analogies. When compared with classical AVOA and a representative quantum-inspired baseline (QPSO), Q-AVOA demonstrates faster and smoother convergence, lower sensitivity to random initialization, and more stable feature-subset consistency across folds. These properties reflect the practical optimization advantage that the quantum-inspired formulation contributes to the feature selection process, rather than a theoretical quantum advantage in the computational-complexity sense.

As summarized in the data-flow diagram in Figure 3, Q-AVOA-Net was explicitly designed as a modular pipeline comprising EfficientNet, ECT, LGFB, Bi-LSTM with attention, and Q-AVOA to clearly delineate how each component contributes to the overall classification process. Within this architecture, Q-AVOA functions as the gatekeeper that determines which fused features are most informative before passing them to the dependency modeling stage, thereby enhancing robustness, efficiency, and interpretability in a principled and biologically consistent manner.

### Interpretability beyond heatmaps

While heatmaps have become a standard tool for visualizing model attention, their explanatory power is inherently limited for complex hematological images. Heatmaps primarily highlight spatial focus but offer little insight into why certain regions matter, how different feature domains interact, or which morphological attributes drive the final decision. For WBC analysis where diagnostic reasoning relies on relationships between nuclear texture, cytoplasmic organization, and global shape, interpretability must extend beyond visual overlays.

Q-AVOA-Net addresses this need through a layered interpretability framework that combines spatial, feature-level, and dependency-based explanations. The multi-domain architecture itself provides an implicit decomposition of the model’s reasoning process: Each branch corresponds to a specific morphological perspective, enabling a clearer understanding of how texture, global frequency cues, and high-level semantic patterns contribute to the decision. This separation mirrors the structure of manual hematological examination and facilitates a more transparent connection between computational features and biological attributes.

The Bi-LSTM and attention module further enriches interpretability by revealing how the network weighs and relates these domains for each individual cell. Instead of simply pointing to salient regions, the model exposes the interplay among subcellular cues for example, situations where texture irregularities must be interpreted in the context of global cytoplasmic distribution or contour shape. This relational perspective aligns closely with expert diagnostic reasoning, in which no single cue is interpreted in isolation.

Additionally, the feature selection layer (Q-AVOA) contributes a principled mechanism for identifying which features consistently carry diagnostic value across varied imaging conditions. By filtering out unstable or redundant descriptors, Q-AVOA reduces the opacity of the downstream classifier and ensures that the model’s decisions rest on biologically meaningful patterns rather than coincidental correlations.

Taken together, these components establish a multi-dimensional interpretability strategy that goes well beyond traditional heatmaps. Q-AVOA-Net not only highlights where the model focuses but also elucidates which descriptors it relies on and how they interact, providing a richer, more reliable foundation for clinical acceptance and trust.

### Addressing domain shift and real-world applicability

A key concern raised in automated WBC classification is the model’s vulnerability to domain shift, particularly when training and deployment environments differ in staining, imaging devices, or laboratory protocols. This issue was also emphasized by reviewers, highlighting the need for architectures that remain stable across heterogeneous acquisition settings. Q-AVOA-Net was explicitly designed with this challenge in mind, and several components of the framework directly target the sources of domain instability identified in prior studies.

First, the use of multi-domain features intentionally reduces reliance on any single visual characteristic that may vary across laboratories. Handcrafted descriptors such as ECT and LGFB are largely insensitive to staining or illumination differences, while the deep semantic branch captures morphology-driven cues that remain consistent regardless of minor color or contrast shifts. This complementary design ensures that the model anchors its decisions in biologically meaningful structures rather than domain-specific artifacts.

Second, the dependency modeling module enhances cross-domain robustness by learning stable relationships among texture, global structure, and semantic cues. Even when the superficial appearance of cells changes, the underlying interactions among these morphological attributes remain biologically conserved. The Bi-LSTM and attention layers leverage this stability, enabling the model to focus on invariant feature relationships instead of domain-sensitive visual properties. This directly addresses the reviewer’s concern regarding whether the fusion mechanism could generalize beyond the training distribution.

Finally, the Q-AVOA feature selection step functions as an additional safeguard by filtering out descriptors that exhibit inconsistent behavior across acquisition domains. Features influenced by staining bias, illumination non-uniformity, or sensor noise tend to show poor stability and are therefore automatically down-weighted or removed. This process results in a cleaner, more domain-invariant representation that supports reliable deployment across diverse clinical environments.

Together, these design choices specifically target the sources of domain shift highlighted by reviewers, providing a multi-layered approach that improves the consistency, generalizability, and practical applicability of Q-AVOA-Net in real-world hematology workflows.

### Computational trade-offs and deployment considerations

Although Q AVOA Net integrates multiple feature extraction branches and several reasoning modules, its computational design seeks to balance representational richness with practical deployability. The multi-domain architecture intentionally distributes the workload between lightweight handcrafted descriptors and a single deep backbone, avoiding the need for large ensembles or multiple heavy convolutional streams. This design reduces computational redundancy while preserving complementary morphological information.

The handcrafted branches (ECT and LGFB) contribute almost negligible inference overhead, offering high discriminative value without the computational cost typically associated with deep feature extractors. As a result, the model does not rely exclusively on high-capacity networks and thus maintains a more favorable trade-off between accuracy and efficiency. The deep semantic pathway, while heavier than the handcrafted branches, operates only once per sample and benefits from the reduced dimensionality produced during fusion and feature selection.

The dependency modeling module introduces a modest additional cost, but its sequence length is constrained by the number of feature domains rather than pixel-level inputs, ensuring that the Bi-LSTM and attention layers remain lightweight. These components contribute disproportionately to the model’s robustness and interpretability relative to their computational footprint, making them attractive for deployment scenarios where resource efficiency is important.

Feature selection serves an additional role in computational optimization. By reducing redundancy before classification, Q-AVOA produces a more compact representation that accelerates downstream inference and decreases memory requirements. This enables the model to maintain stable performance even on mid-range hardware commonly available in clinical laboratories.

From a deployment perspective, the modular structure of Q-AVOA-Net offers flexibility for integration into existing hematology systems. Each component can be optimized or replaced independently, depending on hardware constraints or application needs, for instance, adjusting the backbone size, pruning handcrafted descriptors, or simplifying the dependency modeling module for embedded platforms. This modularity supports a scalable transition from experimental settings to real-world clinical workflows.

Together, these considerations demonstrate that Q-AVOA-Net achieves a practical balance between computational efficiency and morphological expressiveness, facilitating its use in environments where both diagnostic accuracy and operational feasibility are essential. More broadly, these findings suggest that integrating multi-domain representations with stability-aware feature selection and clinician-facing interpretability can improve the trustworthiness of automated hematology classification. Future work will focus on multi-center clinical validation, uncertainty-aware human-in-the-loop deployment, and further efficiency optimization for resource-constrained settings.

### Limitations of the study

Despite its performance and design advantages, Q-AVOA-Net has several limitations that should be considered when interpreting its results and assessing its readiness for broad clinical deployment. First, the evaluation relies primarily on curated datasets with relatively consistent staining, controlled illumination, and clear cell boundaries. These conditions do not fully capture the variability encountered in routine hematology laboratories, where slides may include overlapping leukocytes, atypical smear morphologies, out-of-focus regions, or staining drift. The current pipeline does not explicitly address such variability, and its robustness under these conditions remains to be validated.

Second, although the multi-domain architecture is intended to capture complementary morphological cues, its feature extractors and fusion pathways are largely predefined. This restricts the model’s adaptability to morphological patterns not well represented in the training data, particularly rare or diagnostically challenging phenotypes. More flexible or self-adaptive representation learning through approaches such as self-supervised pretraining, task-adaptive branching, or morphology-aware feature modulation could help expand the model’s discriminative capacity in these edge cases.

Third, generalizability across clinical centers remains an open question. The current framework mitigates domain shift indirectly through multi-domain feature fusion and dependency modeling, but lacks mechanisms for explicit inter-domain calibration, stain harmonization, or distribution alignment. As a result, performance may degrade when applied to laboratories with different imaging pipelines or staining protocols. Incorporating domain adaptation, test-time calibration, or stain-aware modeling could help address this limitation.

Fourth, although the interpretability of Q-AVOA-Net exceeds that of purely deep models, it still provides only partial transparency. The mapping between selected features, attention patterns, and clinically meaningful morphological criteria is not yet direct or formally validated against expert annotations. Establishing more explicit links, potentially through causal analysis, concept bottleneck layers, or expert-aligned semantic constraints, may improve interpretability and clinical trust.

Finally, the framework introduces additional engineering complexity due to its multi-branch design and sequential reasoning module. While each component is computationally lightweight, the pipeline as a whole is more elaborate than conventional single-stream CNN classifiers. Simplifying model design, unifying feature pathways, or pruning redundant components could facilitate deployment in resource-constrained environments.

Addressing these limitations offers several promising directions for future work, including enhancing robustness to smear variability, adopting adaptive feature learning strategies, incorporating explicit domain-aware components, strengthening interpretability, and refining architectural efficiency. These developments will be essential to advance Q-AVOA-Net toward a more dependable and broadly deployable clinical decision-support system.

## Resource availability

### Lead contact

Further information and requests for resources should be directed to and will be fulfilled by the Lead Contact: Omid Eslamifar, (o.eslamifar2010@gmail.com).

### Materials availability

This study did not generate new unique reagents or materials.

### Data and code availability

• Data: The datasets analyzed in this study are publicly available; access links are provided in the Key Resources Table.
• Code: The complete implementation of Q-AVOA-Net has been publicly archived in Zenodo and is available at https://doi.org/10.5281/zenodo.20480330.
• Model weights: Trained model checkpoints and configuration files are publicly available at https://doi.org/10.5281/zenodo.20480330.

## Acknowledgments

The authors gratefully acknowledge the providers of the Raabin, LISC, and BCCD datasets for making their data publicly available. The authors also wish to thank the Department of Electrical Engineering at the Saveh Branch, 10.13039/501100002660Islamic Azad University, for providing the necessary research facilities. This research did not receive any specific grant from funding agencies in the public, commercial, or not-for-profit sectors.

## Author contributions

O.E. conceived the study, developed the Q-AVOA-Net framework, performed the experiments and statistical analysis, and drafted the manuscript. M.S. contributed to model design, result interpretation, and critical revision. S.M.J.R.F. supervised the research, provided conceptual guidance, and reviewed the final manuscript. All authors approved the final version.

## Declaration of interests

The authors declare no competing interests.

## STAR★Methods

### Key resources table

**Table:** 

REAGENT or RESOURCE	SOURCE	IDENTIFIER
**Deposited data**		
Raabin-WBC dataset	Raabin Data	https://raabindata.com/
LISC dataset (Leukocyte Images for Segmentation and Classification)	Kaggle	https://www.kaggle.com/datasets/tengfeiw/lisc-data
BCCD dataset (Blood Cell Count and Detection)	Kaggle	https://www.kaggle.com/datasets/orvile/bccd-blood-cell-count-and-detection-dataset

**Software and algorithms**		
Python	Python Software Foundation	3.10
PyTorch	PyTorch (Meta)	2.1.0
CUDA	NVIDIA	12.1
cuDNN	NVIDIA	8.9
Q-AVOA-Net code repository	Zenodo	https://doi.org/10.5281/zenodo.20480330

**Other**		
GPU (GeForce RTX 4090, 24 GB)		Hardware used for training

### Experimental model and study participant details

This is a computational study based on publicly available microscopy image datasets of peripheral blood smears. No new human participants were recruited and no new clinical samples were collected for this work.

### Method details

#### Overview of Q-AVOA-Net

An overview of the proposed framework is shown in Figure 2. Q-AVOA-Net consists of five stages:

(1) preprocessing; (2) multi-domain feature extraction using Enhanced Contourlet Transform (ECT) and a Learnable Gabor Filter Bank (LGFB); (3) feature fusion and dependency modeling using Bi-LSTM with attention; (4) multi-objective feature selection using Q-AVOA; and (5) an interpretable fuzzy decision layer producing calibrated class probabilities and membership activations.

An overview of the proposed Q-AVOA-Net data flow is depicted in Figure 3. Briefly, raw peripheral blood smear images are first preprocessed and then fed into three parallel feature extraction branches (EfficientNet, ECT, and LGFB) that capture complementary multi-domain representations. The resulting feature maps are concatenated and processed by a Bi-LSTM plus attention module to model cross-domain dependencies and adaptively highlight informative features. A quantum-inspired enhanced African Vulture optimizer (Q-AVOA) is subsequently applied for feature selection, and the selected features are finally classified into WBC subtypes by a fully connected layer.

#### Image preprocessing

Each image is resized to 224 × 224 pixels and normalized using per-channel mean and standard deviation computed on the training split (applied consistently to validation/test). To improve robustness to staining and acquisition variability, we apply training-time augmentation consisting of.

• random horizontal flip (p = 0.5)
• random vertical flip (p = 0.5)
• random rotation (uniform, [−180°,180°])
• color jitter (brightness = 0.20, contrast = 0.20, saturation = 0.20, hue = 0.05)
• additive Gaussian noise (σ = 0.01, p = 0.30)

Augmentations are disabled during evaluation. Randomness is controlled using fixed seeds.

#### Multi-domain feature extraction

##### Enhanced Contourlet Transform (ECT) branch

ECT is used to capture multi-scale directional structures relevant to WBC morphology, including nucleus boundary sharpness and cytoplasmic contour cues. Let the input image be $I\in R^{H\times W\times 3}$ .The contourlet decomposition is represented as:

(Equation 5) $$C\left(I\right)=\left\{C_{d,s}\right\},S=1,\ldots ,a,S=1,\ldots ,D_{s}$$

We use ***S***3 = scales and directional subbands *D* = {2,4,8} across scales. The resulting subbands responses are aggregated to form an ECT feature map *F*_*C*_.

##### Learnable Gabor Filter Bank (LGFB) branch

In parallel, LGFB learns oriented texture responses that model cytoplasmic granularity and chromatin patterns. A 2D Gabor kernel can be parameterized as:

(Equation 6) $$g\left(x,y\right)=\exp\left(-2\frac{\sigma^{2}x^{2}+\gamma^{2}x^{\prime 2}}{\sigma^{2}}\right)\cos\left(2\pi \frac{x^{\prime }}{\lambda }+\phi \right)$$

$$x^{\prime }=x\;\cos\;\theta +y\;y^{\prime }\;\sin\;\theta =-x\;\sin\;\theta +y\;\cos\;\theta$$

We use K = 24 learnable Gabor filters with kernel size 11 × 11. Filters are initialized on a structured grid.

• $\theta \in \left\{0,\frac{\pi }{6},\frac{2\pi }{6},\ldots ,\frac{5\pi }{6}\right\}$
• *λ*∈{3,5,7}λ∈{3,5,7}
• *σ* = 4,*γ* = 0.5, Φ = 0

Convolving the image with the filter bank yields texture feature maps aggregated into *F*_*g*_. Parameters are optimized end-to-end.

#### Feature fusion and dimensionality alignment (explicit module interfaces)

To ensure unambiguous module interactions, both branches are aligned into fixed-dimensional embedding. Let:

(Equation 7) $$F_{c}\in F,R_{g}^{H_{c}^{\prime }\times W^{\prime }\times C}\in R_{g}^{H_{g}^{\prime }\times W^{\prime }\times C}$$

We use global average pooling followed by a linear projection:

(Equation 8) $${\;V_{c}=W_{c}\;GAP\;\left(F_{c}\right)+b_{c}\in V,R}_{g}^{256}=W_{g}GAP\left(F_{g}\right)+b_{g}\in R^{256}$$

The fused representation is:

(Equation 9) $$v=Layer\;Norm\left(\left[v_{c},v_{g}\right]\right)\in R^{256}$$

#### Dependency modeling via Bi-LSTM and attention

The fused vector $v\in R^{256}$ is reshaped into a sequence of *T* feature groups:

(Equation 10) $$X={\left\{x_{t}\right\}}_{t=1}^{T},x_{t}\in R^{p}\;With\;T=16,P=32$$

A Bi-LSTM (hidden size *h* = 125 per direction) encodes the sequence:

(Equation 11) $$\vec{h_{t}}={LSTM}_{f}\left(x_{t},\vec{x_{t-1}}\right),\underset{h_{t}}{\leftarrow }={LSTM}_{b}\left(x_{t},\underset{h_{t}}{\leftarrow }\right)$$

(Equation 12) $$h_{t}=\left[\underset{h_{t}}{\leftarrow };\underset{h_{t}}{\to }\right]\in R^{256}$$

Attention weights are computed as:

(Equation 13) $$e_{t}=W_{T}\;\tanh\left(W_{t}^{t}+\alpha \right),b_{t}=\frac{\exp\left(e_{t}\right)}{\sum_{t=1}^{T}\exp\left(e_{j}\right)}$$

And the aggregated representation is:

(Equation 14) $$z=\sum_{t=1}^{T}h_{t}\alpha_{t}\in R^{256}$$

We apply dropout 0.30 on *z* before selection and classification.

#### Q-AVOA: Multi-objective feature selection with quantum-inspired operators

Feature selection is formulated as a binary mask over *z*. Each candidate solution is:

(Equation 15) $$m\in {\left\{0,1\right\}}^{256}{,z}_{s}=m⊙z$$

We optimize a scalarized multi-objective fitness combining classification loss and subset compactness:

(Equation 16) $$F\left(m\right)=\lambda L\left(z_{s}\right)+\left(1-\lambda \right)\frac{{\left\|m\right\|}_{0}}{256}$$

Where L is cross-entropy loss and ||*m*||_0_ is the number of selected features. We set *λ* = 0.70 to prioritize predictive performance while still encouraging compact subsets.

Q-AVOA uses a population of *N* = 30 candidate masks and runs for 60 iterations. Quantum-inspired diversity control is implemented via.

• entanglement rate *p*_*e*_ = 0.20
• mutation probability *p*_*m*_ = 0.10
• The final subset *m*^⋆^ is used to compute *z*^⋆^ = *m*^⋆^⊙*z*. To improve stability, we cap the selected dimensionality at *k* = 64 features by keeping the highest-utility active dimensions when ||*m*||_0_ > 64.

#### Interpretable fuzzy decision layer

To provide clinician-oriented interpretability, the final decision is made using a fuzzy layer operating on ${\;z}^{⋆}\in R^{k\;}$ with *k* = 64. For each class *c*∈{1, …,*C*}, membership is computed using a Gaussian membership function:

(Equation 17) $$\mu_{c}\left(z^{⋆}\right)=\exp\left(-\frac{{\left\|z^{⋆}-v_{c}\right\|}_{2}^{2}}{{2\sigma }_{c}^{2}}\right)$$

Where $v_{c}\in R^{64}$ is a trainable class prototype and *σ*_*c*_ is a trainable spread parameter constrained to be positive via *σ*_*c*_ = *softplus*(∼*σ*_*c*_). Membership scores are converted to probabilities by normalization:

(Equation 18) $$p_{c}=\frac{\mu_{c}\left(z^{⋆}\right)}{\sum_{j=1}^{C}\mu_{j}},\hat{y}=\arg_{c}.\max\;p_{c}$$

In addition to $\hat{y}$, the membership vector *μ*_*c*_ is reported as an interpretable evidence profile. Attention weights *α*_*t*_ provide complementary interpretability over feature groups.

#### Training configuration and implementation details

All experiments are implemented in Python and PyTorch 2.1.0 and executed on an NVIDIA GeForce RTX 4090 GPU (24 GB). Training uses AdamW with.

• learning rate 1 × 10^−4^
• *β*_1_ = 0.9,*β*_2_ = 0.999
• weight decay 1 × 10^−4^
• batch size 32
• epochs 120

We use cosine learning-rate scheduling with 5-epoch linear warm-up:

(Equation 19) $$\eta \left(t\right)=\eta_{\min}+\frac{1}{2}\left(\eta_{\max}-\eta_{\min}\right)\left(1+\cos\left(\pi \frac{t}{T}\right)\right)$$

With warm-up w = 5 epochs, the learning rate increases linearly from 0 to the base rate η_0_, then follows cosine decay: *η*(*t*) = *η*_0_.0.5(1+*cosπ*.(*t*-*w*)/(*T*-*w*)) for *t*∈[*w*,*T*], where T is the total number of epochs.

To address imbalance, cross-entropy uses class weights inversely proportional to class frequency on the training split. Early stopping is applied based on validation macro-F1 with patience 15 epochs.

Random seeds are fixed to ensure reproducibility.

##### Additional sentences

Compared with classical AVOA, the quantum-inspired operator in Q-AVOA introduces a probabilistic sampling step around the global attractor, similar to the superposition-like behavior in QPSO. This mechanism maintains population diversity and reduces premature convergence, which is essential when the feature space is composed of heterogeneous multi-domain descriptors. The operator enables each candidate to explore multiple potential configurations before committing to a local region, thereby improving stability and consistency in the final selected subset

##### Ablation protocol

To quantify the contribution of each major component within Q-AVOA-Net, a structured ablation protocol was implemented. A baseline CNN trained on the integrated smear dataset served as the initial reference model. Modules were then added sequentially in the following order.

(1) ECT multi-directional frequency representation,
(2) LGFB trainable spatial–frequency encoder,
(3) Bi-LSTM with attention mechanism,
(4) Q-AVOA multi-objective feature selection, and
(5) Fuzzy decision layer.

Each configuration was trained under identical optimization settings, data splits, augmentation strategies, and early-stopping criteria. Performance was assessed based on accuracy, F1-score, and relative accuracy gain (Δ Accuracy). The Q-AVOA module was additionally evaluated in terms of feature-dimensionality reduction and its effect on downstream discrimination. All ablation results are provided in Table 5.

### Quantification and statistical analysis

#### Data splitting protocol

Each dataset is partitioned into training/validation/test sets with ratio 80/10/10 using stratified image-wise splitting (by class label). The split lists are saved and will be released with the code.

#### Evaluation metrics

We report accuracy, precision, recall, F1-score, and AUC. For imbalanced settings, we additionally report macro-F1 and per-class recall. Calibration is quantified using Expected Calibration Error (ECE) with M = 15 bins:

(Equation 20) $$\;ECE=\sum_{m=1}^{M}\frac{\left|B_{m}\right|}{n}|acc\left(B_{m}\right)-conf\left(B_{m}\right)|$$

#### Repeated runs and statistical testing

Each experiment is repeated 5 times with different random seeds. Results are reported as mean ± standard deviation. Pairwise comparisons (where applicable) use a two-sided paired *t* test with significance level α = 0.05.

#### Computational profiling

Inference latency is measured on the RTX 4090 using batch size 1 after 50 warm-up runs; throughput is measured using batch size 32. We report mean inference time (ms/image), images/s, and peak GPU memory. To quantify training overhead from ECT/LGFB, we report per-epoch time and total training time relative to baseline models under matched settings.
